# Genomic stability in response to high versus low linear energy transfer radiation in *Arabidopsis thaliana*

**DOI:** 10.3389/fpls.2014.00206

**Published:** 2014-05-20

**Authors:** Neil D. Huefner, Kaoru Yoshiyama, Joanna D. Friesner, Phillip A. Conklin, Anne B. Britt

**Affiliations:** ^1^Department of Plant Biology, University of California at DavisDavis, CA, USA; ^2^Graduate Program in Genetics, University of California at DavisDavis, CA, USA

**Keywords:** ATM, ATR, double-strand breaks, genomic instability, KU80, LIG4, radiation

## Abstract

Low linear energy transfer (LET) gamma rays and high LET HZE (high atomic weight, high energy) particles act as powerful mutagens in both plants and animals. DNA damage generated by HZE particles is more densely clustered than that generated by gamma rays. To understand the genetic requirements for resistance to high versus low LET radiation, a series of *Arabidopsis thaliana* mutants were exposed to either 1GeV Fe nuclei or gamma radiation. A comparison of effects on the germination and subsequent growth of seedlings led us to conclude that the relative biological effectiveness (RBE) of the two types of radiation (HZE versus gamma) are roughly 3:1. Similarly, in wild-type lines, loss of somatic heterozygosity was induced at an RBE of about a 2:1 (HZE versus gamma). Checkpoint and repair defects, as expected, enhanced sensitivity to both agents. The “replication fork” checkpoint, governed by ATR, played a slightly more important role in resistance to HZE-induced mutagenesis than in resistance to gamma induced mutagenesis.

## INTRODUCTION

The function of a cell, its capacity to proliferate, and ultimately, its viability, are under constant threat from a diverse array of DNA damaging agents and events. Although any lesion can have deleterious effects on an organism, double strand breaks (DSBs) are among the most dangerous types of DNA damage a cell can sustain. The threat such a lesion poses to an organism is influenced by several important factors. The site of damage and the phase of the cell-cycle in which the damage occurs impact both a cell’s response to DSBs and their consequences (Rothkamm et al., 2003; Delacote and Lopez, 2008; Goodarzi et al., 2010). Furthermore, while some DSBs are relatively simple, more complex breaks may arise from secondary damage to the DNA backbone, damage to bases adjacent to the DSB, or the presence of multiple breaks in close proximity. Such variation in DSB complexity also plays an important role in governing the repair of these lesions (Mladenov et al., 2009).

Ionizing radiation (IR) is a particularly potent DNA damaging agent that produces single strand breaks, oxidized bases, abasic sites, and DSBs of varying complexity (McGrath and Williams, 1966; Lehnert, 2008). The efficiency with which an ionizing particle transfers its energy to the medium it passes through, termed linear energy transfer (LET), plays an important role in the nature of the DNA damage caused by exposure to IR (Zirkle et al., 1952). Low LET particles, such as gamma rays and X-rays, deposit their energy inefficiently as they pass through a cell, resulting in widely scattered damage (Costes et al., 2007). Such damage is generally repaired via base excision or nucleotide excision repair in a largely error-free manor (Ward and Chen, 1998). High-LET particles, such as accelerated nucleons or high charge, high energy (HZE) particles, deposit their energy much more efficiently along a discreet track as they pass through matter, resulting in significantly more complex, clustered damage along the track (Goodhead, 1988; Nikjoo et al., 1998, 1999; Sutherland et al., 2001; Costes et al., 2007; Hada and Georgakilas, 2008). In human cell lines, the distribution of DSBs induced by HZE has been shown to lie along a well defined path through the nucleus, whereas gamma-induced DSBs are widely scattered; furthermore, HZE-induced γ-H2AX foci are often so closely packed, that individual foci are difficult to discern (Karlsson and Stenerlow, 2004; Costes et al., 2007). Although life on Earth’s surface is largely protected from exposure to high-LET radiation, it’s hazards are still of significant concern, particularly in the planning of long-duration space missions. Moreover, the damage sustained as a result of exposure to either low or high-LET radiation exhibits similarities with the damage caused by other more common DSB-inducing agents, and may provide insight into the response to and repair of such lesions.

To investigate the immediate and long-term impacts high versus low-LET radiation have on plants, we employed the various DNA repair-deficient and cell-cycle checkpoint-deficient lines in the model plant *Arabidopsis thaliana*. ATM (ataxia-telangiectasia, mutated) and ATR (ATM and Rad3-related), members of the phosphoinositide-3-kinase-related protein kinase (PIKK) family, play important roles in governing the transcriptional response to DNA breakage and in the induction of cell-cycle arrest (Durocher and Jackson, 2001; Khanna and Jackson, 2001; Sancar et al., 2004; Culligan et al., 2006). While ATM and ATR have been shown to recognize DSBs and stalled replication forks respectively, it is clear that both kinases play important roles in the IR-induced DNA-damage response (Chen et al., 1999; Gatei et al., 2000; Abraham, 2001; Friesner et al., 2005; Ismail et al., 2005). Given the distinct nature of the lesions these proteins target, we were interested in the role these checkpoint genes play in responding to DNA damage of variable complexity as induced by exposure to high versus low-LET radiation. Lines lacking *DNA ligase IV (LIG4)* or *KU70*, important players in the canonical nonhomologous end-joining (NHEJ) repair pathway, were also utilized to probe the importance of NHEJ and alternative repair pathways in the response to damage done by high and low-LET radiation.

## MATERIALS AND METHODS

### PLANT LINES

The following DNA repair-deficient and cell-cycle checkpoint-deficient alleles were used in our root growth assay: *atm-1* (Garcia et al., 2003), *atr-3* (Culligan et al., 2004), *lig4-1* (Friesner and Britt, 2003), and *ku80-1* (Friesner and Britt, 2003); all of which are in the *Ws* ecotype.

For our sectoring assay, we searched the Arabidopsis Information Resource (TAIR) database to identify alleles that might serve as an albino marker. The *APG3* gene (*a*lbino/*p*ale *g*reen mutant 3), located near the telomere of chromosome III, is essential in chloroplast development (Motohashi et al., 2007). Seedlings deficient for APG3 arrest development shortly after germination; as the name implies, cells deficient for APG3 lack pigment and are easily distinguished from those that retain a wild-type copy of the *APG3* gene. A line harboring a T-DNA insertion in *APG3* was ordered from the Arabidopsis Biological Resource Center (Syngenta stock “CS16118”; McElver et al., 2001). This particular allele, *apg3-2*, was selected both because the T-DNA construct used in generating this particular line imparts resistance to the herbicide Basta, and because of its clear and consistent albino phenotype; the presence of the Basta resistance (*BAR*) gene in this construct afforded us the ability to select for just those plants carrying the albino marker. To introduce the APG3 albino marker into our repair-deficient and checkpoint-deficient lines, we tried where possible, to choose alleles that did not already carry the *BAR* gene. We crossed *apg3-2* with *atm-1* (Garcia et al., 2003), *atr-2* (Culligan et al., 2004), *lig4-3* (Hefner et al., 2003, 2006), and *ku80-1* (Friesner and Britt, 2003); the ecotype of *apg3-2* and *atr-2* is *Col*, that of *atm-1* and *ku80-1* is *Ws*, and that of *lig4-3* is *Ler*.

### IONIZING RADIATION

High-LET radiation treatments were administered at the NASA Space Radiation Laboratory (NSRL) at Brookhaven National Laboratory (BNL) (Upton, NY) using accelerated ^56^Fe nucleons with a beam size diameter of 20 cm and a dose rate of 7 Gy min^-^^1^. Following irradiation, samples remained at the NSRL facility for approximately 30 m until deactivated.

Low-LET, gamma radiation treatments were carried out at BNL using a ^137^Cs source in the Controlled Environment Radiation Facility at a dose rate of up to 6 Gy min^-^^1^. A subset of the gamma radiation treatments done on samples for the albino sectoring assay were carried out using an alternate ^137^Cs source (Institute of Toxicology and Environmental Health, University of California, Davis, CA, USA) with a dose rate of 7 Gy min^-^^1^.

### PREPARATION OF SAMPLES USED IN ROOT GROWTH AND SECTORING ASSAYS

Four to six days prior to irradiation, seeds used in the root growth assay were surface sterilized using a 20% bleach solution; sterilized seeds and seeds used in the sectoring assay were aliquoted, suspended in ddH2O, and stored at 4°C. Seeds were shipped overnight, on ice, to BNL where they were again stored at 4°C until the time of IR treatment. Samples were irradiated (See “*Ionizing Radiation*” above) with the doses indicated in the text and figures. Following treatment, the samples were repacked on ice and shipped overnight to the University of California, Davis. Upon arrival, seeds used in the sectoring assay were sown on soil (Sunshine Mix #1; Sungro, Bellevue, WA, USA) at a density of ~0.2 seeds cm^-^^2^ and placed in the growth chamber under clear plastic domes to maintain high humidity; seeds used in the root growth assay were sown on 1× nutrative MS (Sigma-Aldrich, Saint Louis, MO, USA) Phytoagar (PlantMedia, Dublin, OH, USA) plates, pH 5.9, and placed vertically in the growth chamber. Seeds were grown under a simulated 16 h day/8 h night cycle using light from cool-white lamps (100-150 μmol m^-^^2^ s^-^^1^) filtered through Clear UV-filtering Protect-O-Sleeves (McGill Electrical Product Group, Rosemont, IL, USA). Plastic domes were partially removed from seeds used in the sectoring assay 3 days after sowing and fully removed after 5 days.

### MEASUREMENT OF ROOT LENGTH

Digital images of the plated seeds were taken eight days after transfer to the growth chamber. The length of the primary root was determined using the public domain, image-processing program, ImageJ.

### QUANTIFICATION OF APG3 SECTORS

Because the *apg3-2* allele imparts resistance to the herbicide Basta, we were able to significantly reduce the number of plants it was necessary to screen for sectoring. Roughly 2 w after sowing, the number of healthy seedlings was determined. Plants were then treated with Basta (Finale, AgrEvo Environmental Health, Montvale, NJ, USA) and the number of resistant plants was determined in the following days. Resistant plants were scored for the presence of albino sectors approximately 3 weeks after sowing. The leaves of each plant were gently manipulated in order to inspect underlying leaves. Sectors were white or pale green in color, could be traced toward or along the petiole, and exhibited a relatively clear and well-defined boundary between the sector and the neighboring tissue; leaves that were clearly unhealthy or grossly deformed were not scored for sectors. While the number of Basta resistant plants might have been used to calculate such a frequency directly, the fact that In the case of the *atm-1* mutant, this insertion allele also carries Basta resistance, and so seedlings in the next generation, after selection for Basa resistance, would be expected to segregate 2 *apg3-2* het:1 *APG3-2*^+^/^+^. To correct for this, seeds were collected from a heterozygous parent, and the frequency of hets for *apg3-2* was corrected for in the next generation. To estimate the frequency of sectoring in the progeny of heterozygous *APG3*^±^ plants, we divided the number of plants with a sector by two-thirds the total number of healthy seedlings present 2 weeks after sowing.

## RESULTS AND DISCUSSION

### THE BIOLOGICAL EFFECTIVENESS OF HZE ^56^Fe PARTICLES IS APPROXIMATELY 3-FOLD GREATER THAN THAT OF ^137^Cs GAMMA RAYS WITH RESPECT TO INHIBITION OF PRIMARY ROOT GROWTH

**FIGURE 1:**
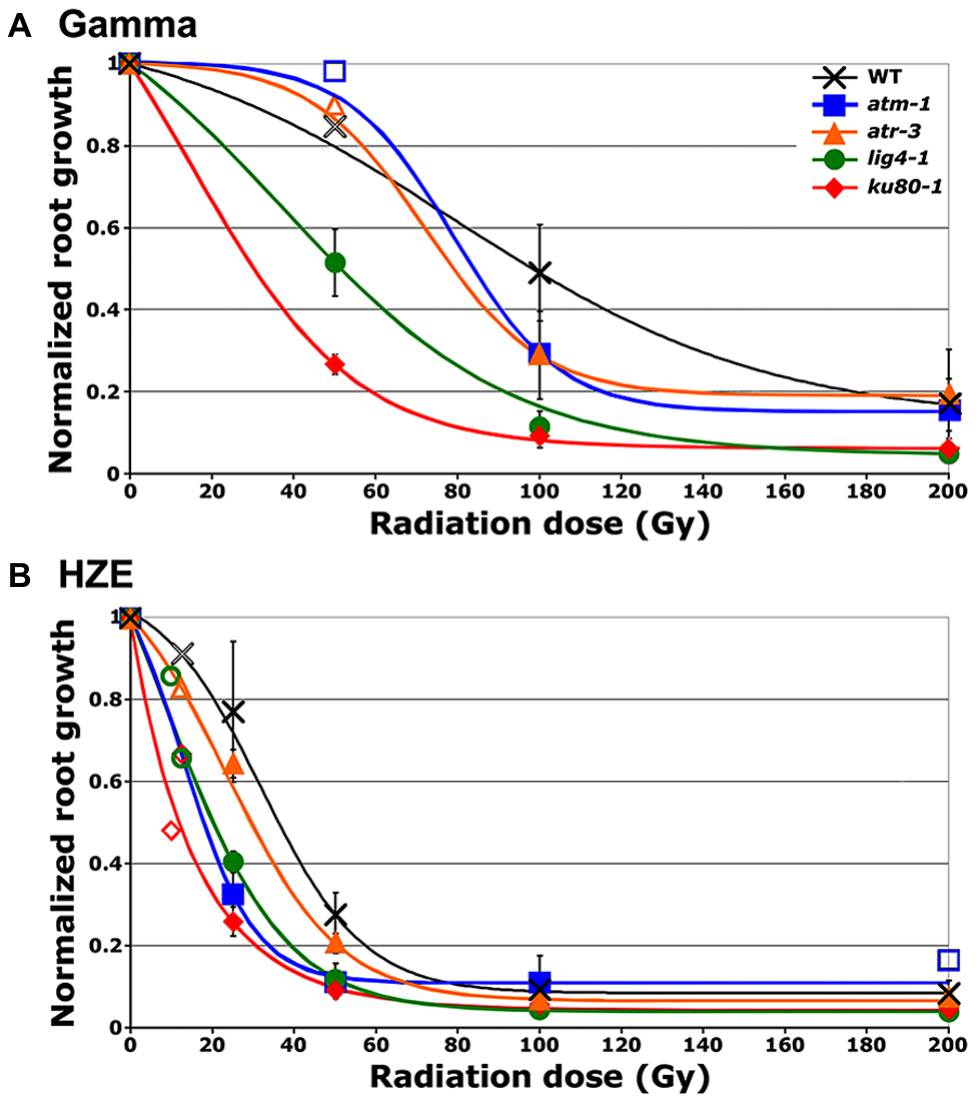
**Relative root growth of irradiated seeds 8 days after treatment. (A)** Gamma treatment. **(B)** HZE treatment. Data points reflect results from three (*n* = 3) biological replicates in the case of *atm*, and *atr* and four (*n* = 4) biological replicates in the case of wild-type, *lig4* and *ku80* (Doses for which only a single biological replicate was performed are displayed as hollow data points.). Rootlengths from an average of 29 seedlings were scored per line, per treatment, per replicate. Lines represent interpolation of the data, fit to a sigmoid curve. Error bars depict standard error of the mean, as calculated and plotted by Microsoft Excel.

To test the relative impact of high- versus low-LET radiation on root growth, a process governed by both cell division and cell expansion, seeds were irradiated with either high-LET ^56^Fe particles (HZE) or low-LET ^137^Cs gamma rays. The length of the primary root 8 days after planting (DAP), relative to the length in unirradiated seeds, is shown in **Figure 1**. Consistent with previously published data, exposure to increasingly higher doses of gamma radiation results in increased inhibition of root elongation (Jiang et al., 1997). A similar, though more pronounced trend is observed in seeds exposed to HZE. At doses of 100 Gy HZE or higher, root elongation appears to be almost completely inhibited in wild-type lines, though seeds irradiated at such doses are still capable of germination, indicating that the treated embryos remain alive.

Relative biological effectiveness (RBE), defined as the absorbed dose of radiation of a standard type (e.g. gamma) divided by the absorbed dose of radiation type “*x*” that causes the same amount of biological damage, offers a means of comparing how damaging different types of radiation are, given the same amount of absorbed energy; the larger the RBE for a type of radiation, the more damaging the radiation per unit energy deposited (Failla and Henshaw, 1931). The RBE of HZE versus gamma radiation was estimated by interpolating the dose response data to determine the dose at which the length of the primary root is reduced to 37% that of the untreated control (ID_1__/e_; **Table 1**). In the case of both our wild-type and mutant lines, the effect of HZE on primary root growth is significantly greater than that of gamma rays. The biological effectiveness of HZE versus gamma in our repair-deficient lines is slightly lower than observed in wild-type. Somewhat more variance is observed in our checkpoint-deficient lines. Lines deficient for ATM exhibit a slight increase in root sensitivity to HZE versus gamma radiation relative to wild-type (RBE = 3.63 versus 2.97), while lines deficient for ATR exhibit a slight decrease in their relative HZE sensitivity (2.09). Whether this shift in root hypersensitivity is a function of cell death resulting from a failed checkpoint induction, or from prolonged cell-cycle arrest is unclear from the root growth data.

**Table 1:** Relative biological effectiveness of HZE versus gamma radiation with respect to root hypersensitivity.

	ID_1/e_ (Gy)	
Line	^*56*^Fe-HZE	^*137*^Cs-γ	RBE (ID*γ* / ID_HZE_)
Wild-type (Ws)	43.9	130.1	2.97
*atm-1*	22.8	82.9	3.63
*atr-3*	39.0	81.6	2.09
*lig4-1*	27.1	65.8	2.43
*ku80-1*	19.0	40.2	2.12

*ID_1/e_ = Dose required to inhibit growth of primary root to 37% that of the untreated control.*

### GENOMIC INSTABILITY IN SEEDS EXPOSED TO HZE ^56^Fe PARTICLES IS GREATER THAN THAT OBSERVED IN SEEDS EXPOSED TO ^137^Cs GAMMA RAYS

**FIGURE 2:**
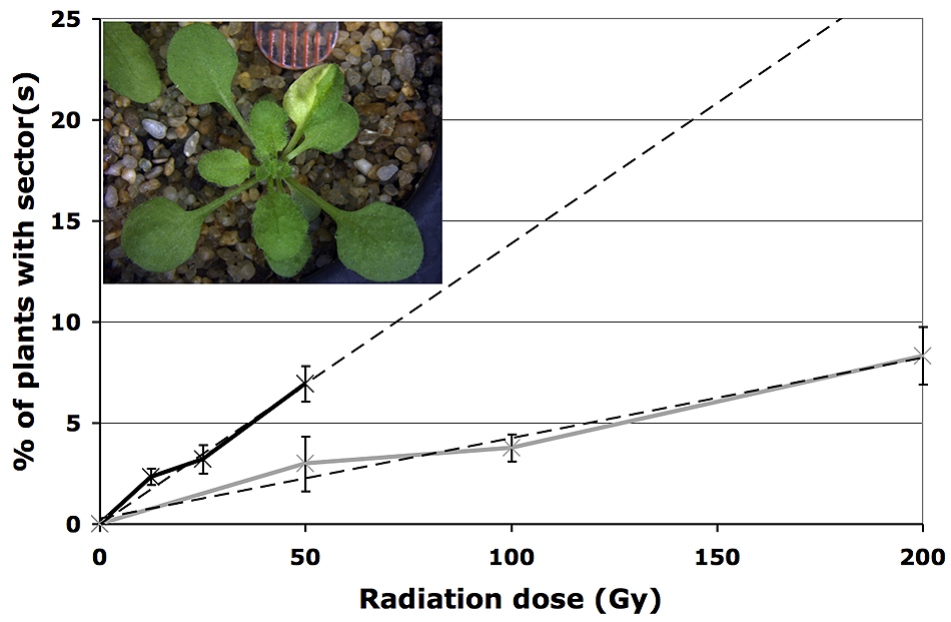
**Loss of heterozygosity following exposure to radiation.** Loss of heterozygosity in wild-type plants treated with HZE (black line) and gamma (gray line) radiation. Data points represent the mean of three biological replicates; dashed lines depict linear regressions of datasets. Error bars depict standard deviation, as calculated by standard statistical methods in Mircosoft Excel. Inset: example of an IR-induced albino sector.

While the root hypersensitivity assay indicates that exposure to HZE has a more pronounced effect, per Gy on the growth of seedlings than does exposure to gamma radiation, it fails to shed much light on long-term effects on genomic stability. In order to address the impact HZE and gamma radiation have on the integrity of the genome, we employed a sectoring assay to test for loss of heterozygosity (LOH) in treated seeds (Preuss and Britt, 2003). An albino marker gene (*apg3*) near the tip of chromosome III was introduced to a series of DNA repair defective lines and checkpoint defective lines. While seedlings homozygous for the albino marker arrest and die shortly after germination, untreated seedlings heterozygous for the marker appear phenotypically identical to wild-type. Loss of the wt allele marker in a heterozygous single cell, whether as a result of anueploidy, loss of the distal portion of the chromosome, or mutation of the wild-type allele, followed by production of mutant cell files via cell division, results in the production of an albino sector (**Figure 2**, inset) The frequency with which these sectors occur within a population provides a measure of genomic stability (Yoshiyama et al., 2009).

As expected, under normal conditions, genomic stability in wild-type plants appears quite high. To obtain an estimate of the rate of spontaneous LOH at the *APG3* locus in wild-type plants, 900 untreated plants were scored for the presence of an albino sector. Of the 900 plants scored, none exhibited the presence of a sector, suggesting a rate of spontaneous LOH per plant for this particular allele of less than 0.1%.

**FIGURE 3:**
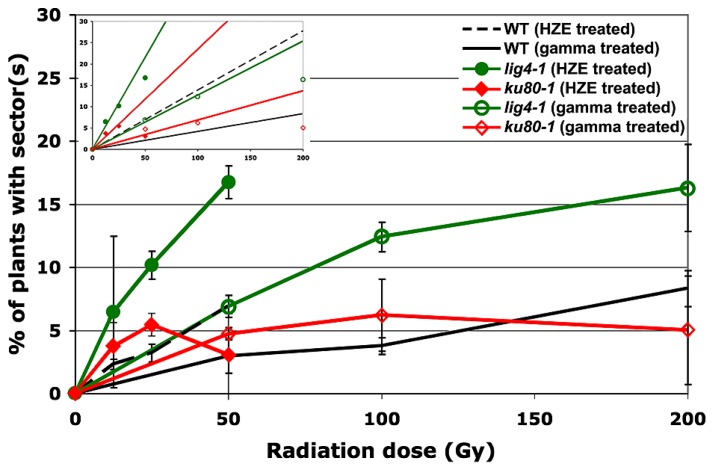
**Loss of heterozygosity in repair-deficient plants in response to HZE and gamma radiation.** Data points represent the mean of three biological replicates. An average of 216 plants were scored per line, per treatment, per replicate. Error bars depict standard deviation, as calculated by standard statistical methods in Microsoft Excel. Inset: Linear regressions for each dataset. Note: as described in the text, the 50 Gy HZE and 200 Gy Gamma data points were excluded from the regression analysis.

In seeds treated with low does of either HZE or gamma radiation, sectoring increases with dose in a roughly linear, positive fashion (**Figure 2**). However, plants treated with higher doses are very small, and exhibit decreasing frequencies of sectoring/plant, perhaps simply because fewer cells are sampled per plant. Sectoring frequency in seeds treated with ^56^Fe particles was consistently higher than that observed in seeds treated with gamma rays; these results are consistent with the observation that in human cells, clustered damage generated by Fe ions leads to increases in chromosome breakage and genomic instability (Asaithamby et al., 2011). To obtain an estimate of the biological effectiveness of HZE versus gamma radiation, in the context of long-term genomic stability, linear regressions were generated from the sectoring data (**Figures 2** and **3**, inset). Regressions were constrained such that they passed through the origin, and the ratio of the slopes from the HZE and gamma datasets was determined. In wild-type plants, a 3.33-fold increase in sectoring was observed in seeds treated with HZE as compared to those treated with gamma rays (**Table 2**). In the case of *lig4* and *ku80*, the 50 Gy gamma and 200 Gy HZE datapoints were omitted in the regression analysis due to a drop-off in sectoring at higher doses as discussed in the following section.

**Table 2:** Relative biological effectiveness of HZE versus gamma radiation with respect to LOH.

Slope of Linear Regression “*y* = mx” (fraction of *APG3*^±^ plants with visible sector/Gy)			
Line	m (^56^Fe-HZE)	m (^137^Cs-γ)	RBE (m_HZE_/m*γ)*
Wild-type	0.1389	0.0417	3.33
*atm-1*	0.2626	0.1024	2.56
*atr-2*	0.3079	0.0784	3.93
lig4-3	0.4292	0.1268	3.38
ku80-1	0.2343	0.0686	3.42

*Calculations were made as described in the text, where RBE of HZE relative to gamma is taken as the ratio of slopes (m_HZE_/m*γ*) for the linear regressions of the sectoring versus dose datasets forced through the origin.*

### LINES DEFECTIVE IN DNA DSB REPAIR EXHIBIT A DECREASE IN GENOMIC STABILITY

Given the significant roles LIG4 and the KU70/KU80 heterodimer play in NHEJ (Friesner and Britt, 2003), we sought to determine the importance of these factors in maintaining genomic stability during the repair of IR-induced DSBs. As in the case of wild-type, at low doses, our *lig4* and *ku80* lines exhibited a roughly linear increase in the frequency of sectoring with exposure to increased levels of radiation; however, unlike our wild-type line, a significant drop-off in sectoring was observed at higher doses of either HZE or gamma radiation (**Figure 3**). Again, we believe this is due to the very small size of the repair-defect plants after irradiation at these doses.

Of the mutant lines tested, plants lacking LIG4 exhibited the highest rate of sectoring following exposure to IR (**Table 2**). Although research has demonstrated that DSBs can be rejoined in the absence of LIG4, its role as the primary ligase involved in NHEJ is well documented (Grawunder et al., 1998; Tsukamoto and Ikeda, 1998; Junop et al., 2000; Friesner and Britt, 2003; van Attikum et al., 2003; Huefner et al., 2011). The increased rate of LOH in *lig4* is almost certainly the result of persistent DSBs present during cell division; whether the structure of these breaks precludes other ligases from rejoining the broken ends, or the ends are repaired in a more error-prone fashion is unclear. KU80, another important component of NHEJ, also appears to be involved in maintaining genomic stability following exposure to IR. Other work has shown that while the KU complex plays an important role in governing the size of deletions and insertions at DSB repair sites and in stabilizing broken ends prior to ligation, end-joining can still occur in its absence (Ma et al., 2003; Boboila et al., 2010; Huefner et al., 2011). It is likely, therefore, that a relatively large portion of the broken DNA ends generated by IR are ultimately rejoined in our *ku80* line resulting in the more moderate increase in sectoring observed in *ku80* plants as compared to *lig4* plants.

### THE RELATIVE IMPORTANCE OF ATM AND ATR IN MAINTAINING GENOMIC STABILITY DIFFERS IN RESPONSE TO HZE VERSUS GAMMA RADIATION

**FIGURE 4:**
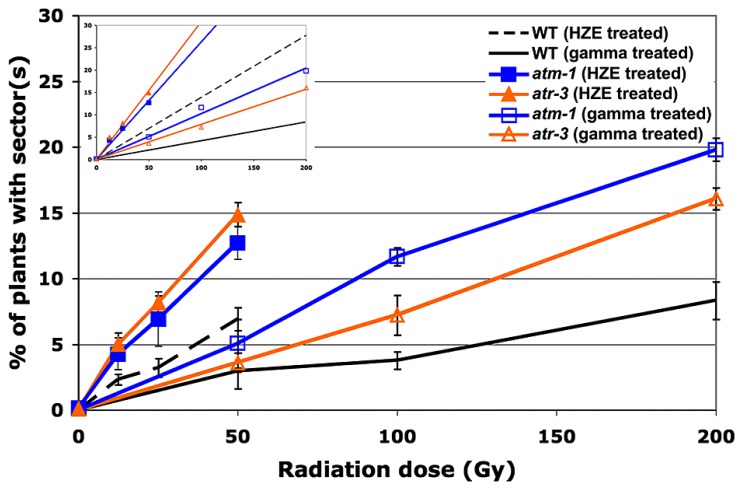
**Loss of heterozygosity in cell-cycle checkpoint-deficient plants in response to HZE and gamma radiation.** Data points represent the mean of three biological replicates. Error bars depict standard deviation, calculated as before via Microsoft Excel. Inset: Linear regressions for each dataset.

As with our DNA repair-deficient lines, a significant increase in sectoring was observed in our checkpoint-deficient lines relative to wild-type (**Figure 4**). Such an increase is consistent with the role ATM and ATR play in governing the DSB driven, early G2/M phase arrest. Failure to properly arrest cells in response to DSBs could result in the presence of persistent DSBs during cell division, thereby leading to aneuploidy and LOH (Garcia et al., 2003; Culligan and Britt, 2008). ATM and ATR function synergistically in response to IR-induced DNA damage, functioning in some ways redundantly and in other ways distinctly to activate downstream targets, trigger cell-cycle arrest, and help drive DNA repair. In light of the fact that ATM is thought to respond directly to DSBs via interaction with proteins associated with the breaks (Bakkenist and Kastan, 2003; Lee and Paull, 2005), whereas ATR is thought to respond to the presence of persistent single-stranded DNA (ssDNA) (Burrows and Elledge, 2008; Cimprich and Cortez, 2008), we were interested in whether ATM and ATR function differently in maintaining genomic stability in response to the damage caused by HZE versus gamma radiation.

*atm-1* seeds exposed to gamma radiation exhibit a 2.5-fold increase in the frequency of sectoring as compared to wild-type (calculated from the slopes reported in **Table 2**). A more moderate increase in sectoring, 1.9-fold, was observed in our *atr* line, suggesting that while both ATM and ATR play a role in maintaining genomic stability in response to gamma radiation exposure, ATM is of greater relative importance. In seeds exposed to HZE particles, an increase in sectoring was again observed in both *atm* and *atr* relative to wild-type. Fold increases of 1.9 and 2.2 were observed for *atm* and *atr* respectively, indicating a reversal in the relative importance of ATM and ATR in mitigating LOH in HZE versus gamma treated seeds, although these differences are small. Quantification of the RBE of HZE versus gamma, in terms of genomic stability, in our checkpoint-deficient lines demonstrates a decrease in the relative importance of ATM in response to HZE radiation as compared to wild-type (2.6 and 3.3, respectively), and an increase in the relative importance of ATR (3.9). Based on the LOH data, it appears that ATR plays a more important role in responding to the complex, clustered damage induced by HZE particles than it does in responding to damage induced by exposure to gamma radiation.

Given that ATR responds to persistent ssDNA, one possible explanation for the increased importance ATR plays in response to HZE treatment, is that more ssDNA is produced directly upon exposure to HZE particles. Multiple nicks to the DNA backbone in close proximity to one another may produce significant stretches of ssDNA in conjunction with DSBs not typically present in breaks generated by low-LET radiation. Alternatively, ssDNA may also be produced secondarily in HZE treated cells as the cell attempts to repair the clustered damage via resection of damaged ends or excision of damaged nucleotides. In cells deficient for ATR, imposition of cell-cycle arrest in HZE treated cells may be abrogated or delayed, resulting in enhanced loss of heterozygosity in cells that divide before the damage can be resolved. This may also provide insight into the observation that relative to both wild-type and atm, root growth in atr is slightly more resistant to HZE treatment, given that affected cells, while incurring greater damage to the genome, may still be capable of cell division and expansion.

## CONCLUSION

Our results demonstrate a clear difference in the sensitivity of *Arabidopsis* to high- versus low-LET radiation. Data from both our root hypersensitivity and LOH assays indicate that the RBE of HZE radiation is between two and four times that of gamma radiation. The increased sensitivity of plants to HZE suggests that not only the quantity, but also the complexity of DSBs induced by IR play an import part in determining the efficiency and accuracy of DNA repair. A significant decrease in the genomic stability of KU80-deficient and LIG4-deficient lines in response to both HZE and gamma radiation reflects the importance of C-NHEJ in the repair of both simple and complex DSBs.

While it is unclear what additional factors may be unique to or of special importance in the repair DSBs induced by one class of radiation versus another, it is apparent that the relative importance of ATM versus ATR shifts in response to HZE versus gamma radiation. The increased relative importance of ATR versus ATM in responding to damage induced by HZE suggests that treatment with high-LET radiation results, either directly of indirectly, in a significant increase in the amount of ssDNA in the cell. Given the differences in the composition of DNA damage induced by HZE and gamma radiation, it will be interesting to determine what other differences exist in a cell’s response to both types of IR. Differences in response at the transcriptomics level are addressed in the accompanying paper.

## Conflict of Interest Statement

The authors declare that the research was conducted in the absence of any commercial or financial relationships that could be construed as a potential conflict of interest.
